# Hospital admissions for stress-related presentations among school-aged adolescents during term time versus holidays in England: weekly time series and retrospective cross-sectional analysis

**DOI:** 10.1192/bjo.2021.1058

**Published:** 2021-11-19

**Authors:** Ruth Blackburn, Omotomilola Ajetunmobi, Louise Mc Grath-Lone, Pia Hardelid, Roz Shafran, Ruth Gilbert, Linda Wijlaars

**Affiliations:** UCL Institute of Health Informatics, University College London, UK; UCL Institute of Health Informatics, University College London, UK; UCL Institute of Health Informatics, University College London, UK; Population, Policy and Practice Research and Teaching Department, UCL Great Ormond Street Institute of Child Health, University College London, UK; Population, Policy and Practice Research and Teaching Department, UCL Great Ormond Street Institute of Child Health, University College London, UK; Population, Policy and Practice Research and Teaching Department, UCL Great Ormond Street Institute of Child Health, University College London, UK; Population, Policy and Practice Research and Teaching Department, UCL Great Ormond Street Institute of Child Health, University College London, UK

**Keywords:** Adolescents, stress, administrative data, mental health, school

## Abstract

**Background:**

Schools are a potential stressor for adolescents and may contribute to emergency hospital admissions.

**Aims:**

We describe rates of stress-related presentations (SRPs) among school-aged adolescents (11–17 years) during school terms and holidays, and explore differences by age and gender.

**Method:**

Using national administrative hospital data, we defined an SRP as an emergency hospital admission with a primary diagnosis related to pain, psychosomatic symptoms (e.g. fatigue) or mental health problems, or with self-harm indicated in any diagnostic position. We estimated incidence rate ratios for weekly SRPs in term time versus holidays from 2014–2015 to 2017–2018, using negative binomial regression models, stratified by age and gender. We estimated the cumulative incidence of any SRP between 11 and 17 years by analysing prior hospital admission histories of adolescents with an SRP in 2017–2018.

**Results:**

Over the 4-year study period, 305 491 SRPs in 171 013 school-aged adolescents accounted for 31% of emergency admissions for this group. SRPs were predominantly for mental health problems or self-harm (38%), or pain (35%). Weekly admission rates for SRPs were higher in term time than holidays for all ages (age-specific incidence rate ratios were 1.15–1.49 for girls and 1.08–1.60 for boys). Rates were highest for girls aged 14 and 15 years. The estimated cumulative incidence of any SRP between 11 and 17 years was 7.9% for girls and 4.1% for boys.

**Conclusions:**

Hospital admissions for SRPs are common among adolescents, affecting around two girls and one boy in every classroom. Higher rates in term time than holidays suggest that school factors may contribute.

Schools can be both a potential source of support and a stressor for school-aged adolescents.^1–3^ Although schools can offer structure, stability and social support networks to many students, this is not the experience for all children.^4^ Teacher interactions, self-perception of one's own academic abilities, academic stress (including not keeping up with peers) and peer relationships or victimisation have been cited as sources of distress.^4,5^ Manifestations of stress can be emotional, including feelings of frustration, irritability, anxiety, low mood, alienation or failure; behavioural, including disruptive or aggressive behaviour, substance misuse or self-harming; and physiological, including psychosomatic symptoms and signs.^4^ Symptoms of pain, such as abdominal pain or headache, which are medically unexplained, are frequently reported by adolescent girls and boys.^6^ Physiological manifestations of stress also include cardiovascular or gastrointestinal symptoms.^6–8^ These symptoms are common and often coexist. For example, in a national self-report survey of 9969 11- to 17-year-olds conducted in schools in Ireland, up to 44% of girls and up to 42% of boys reported weekly psychosomatic symptoms (most frequently irritability, headache and stomach ache), with considerable variation in the prevalence of symptoms by age.^7^ Gender is also an important factor in stress-related symptoms, with girls reporting more psychological distress than boys.^6,7^ An overview of studies in 73 countries reported higher rates of psychological distress in girls as a ubiquitous finding.^9^ This gender gap was stronger in higher income and more gender-equal settings. Poorer socioeconomic status, family conflict and parental mental health problems have been associated with self-reported and medically presenting stress-related symptoms.^7,8^

Symptoms of distress affect learning, which in turn leads to difficulties keeping up at school and further stress.^1,4^ In this cycle of stress and distress, mental health problems may be an outcome or a common cause. For example, school failure is a predictor of depression and self-harm,^10,11^ and adolescents with mental health problems have lower school attainment, are more likely to be victimised and have higher rates of stress-related symptoms and behaviours.^1,7,8,11–13^ In some adolescents, stress-related presentations (SRPs) signal underlying mental health problems. A nationally representative survey in England in 2017 reported that 14% of 11- to 16-year-olds, and 17% of 17- to 19-year-olds have at least one diagnosable mental health problem.^14^ Interventions to improve mental health in schools is a government priority, given the high frequency of mental health problems, the impact on learning and the importance of early intervention to improve long-term outcomes.^15–17^ However, there has been less focus on the full range of SRPs, how frequently these present to healthcare and to what extent they can be reduced by changes to the school environment.^12,13^

## Aims

Our aim was to assess the contribution of the school environment to SRPs by comparing rates of emergency admissions for stress-related symptoms or signs during the school term and holidays. Higher rates of SRPs during term time could identify groups of adolescents who might benefit from supportive educational or health interventions, or from less stressful school environments. In this study, we focused on adolescents with SRPs severe enough to warrant emergency admission to hospital, and analysed a broad spectrum of SRPs, including unexplained pain, other non-specific symptoms (such as fatigue), health behaviours relating to self-harm and mental health problems. We also examined whether differences in SRPs varied by gender, age and over time.

## Method

### Data source, population and time period

We used the Health Episode Statistics Admitted Patient Care (HES APC), an administrative hospital database, which captures all admissions to National Health Service (NHS) hospitals in England and collects information on diagnoses and procedures by using standardised codes that are recorded by clinical coders based on patient discharge records. Up to 20 diagnostic codes and 24 operation codes can be recorded for each admission.^18^

The study population included all adolescents resident in England who were of secondary school age (i.e. aged 11–17 years) and had an emergency (i.e. unplanned) hospital admission between 1 September 2014 and 31 August 2018 (*N* = 591 576). We excluded adolescents who died in hospital because retrospective clinical coding at the time of hospital discharge may systematically differ for patients who died compared with those who were discharged alive. As one of the study objectives was to explore gender differences in rates of SRPs, we also excluded adolescents with unrecorded gender and any pregnancy-related admissions. The final study sample included 571 388 adolescents (see Supplementary Table 1 available at https://doi.org/10.1192/bjo.2021.1058 for the full data flow diagram).

In England, the secondary school year comprises three terms (autumn, spring and summer), and school holidays include a half-term break every term and longer end-of-term holidays (Easter, Christmas and summer). For state-funded secondary schools, which around 94% of school children in England attend,^19^ term and holiday dates are set by local authorities, with only minor variation across the country. For this study, we defined term time and holidays for the academic years 2014–2015 to 2017–2018 based on a sample of school timetables published online by local authorities in England (see Supplementary Table 2 for exact dates).

### Outcomes

We defined an SRP as an emergency admission in which the primary diagnosis was a stress-related code or where self-harm was recorded as a non-primary diagnosis. Admissions were not limited to mental health or psychiatry specialties: all emergency admissions, regardless of specialty, were included in this analysis. Hospital transfers or admissions within 1 day of discharge were treated as a continuation of the previous admission.^20^

To identify SRPs, we developed a list of codes for presentations indicating stress-related signs and symptoms in school-aged adolescents, by iterative mapping of clinical conditions reported in the research literature^7,21,22^ to the ICD-10 and clinician review by a clinical psychologist (R.S.) and paediatrician (R.G.). The full code list is given in Supplementary Table 3. We included codes reflecting unexplained pain, potentially psychosomatic symptoms, and those reflecting mental health problems or self-harm behaviours. Pain presentations comprised codes indicative of symptoms of stress such as abdominal pain (R10) or headaches (R51X). Admissions with a primary diagnostic code of abdominal pain (R10) for which a subsidiary code indicating a medical or surgical cause was recorded for the same admission were also excluded (see Supplementary Table 4 for further details). Potentially psychosomatic symptoms included unexplained cardiovascular or respiratory distress, fainting, fatigue or malaise. Mental health and self-harm presentations comprised codes indicative of anxiety (e.g. ICD-10 code F320); mood disorders, including single or recurrent episodes of depression (e.g. ICD-10 codes F32 and F33); and substance misuse (ICD-10 codes F10–F19). Adolescents presenting with self-harm, poisoning or drug/alcohol misuse were identified either through a code indicating evidence of intent (intentional self-harm: ICD-10 codes X60–X84; intentional self-poisoning: ICD-10 code Z642), or a diagnosis reflective of injury or poisoning (e.g. ICD-10 codes S50–S60; superficial injury of forearm/wrist and hand) in combination with a history of self-harm (personal history of self-harm: ICD-10 code Z915).^22^ The three categories of SRPs (pain-related, other somatic and mental health and behavioural) are mutually exclusive, as detailed in Supplementary Table 3.

To provide a comparison group of other emergency presentations, we identified admissions for accidental injuries. We assumed that accidental injuries would not be related to school stress, but acknowledge that rates may be higher during term time than holidays because of circumstances such as participation in school sports. Accidental injuries were defined as published elsewhere,^23^ through ICD-10 codes for accidents (V), accidental injuries (W0–W9) and exposures recorded in any diagnostic position (X0–X5) (see Supplementary Table 5 for further details). Admissions where these accidental injury codes were identified were not categorised as SRPs, such that the two groups were mutually exclusive.

### Covariates

HES APC includes patient demographic information, including gender, age, ethnic group and area-level measures of deprivation. We categorised ethnic group as White, Black, Asian or other ethnicity. We calculated the length of hospital stay based on the recorded admission and discharge dates. To capture indicators of healthcare history, we created indicators for any unplanned hospital admission, adversity-related admissions (related to self-harm or drug-, alcohol- or violence-related injuries) or chronic conditions from 2010–2011 to 2016–2017 (i.e. the preceding 7-year period). We used existing phenotypes to identify chronic conditions and adversity-related admissions, which are published elsewhere.^23,24^

### Statistical analyses

We calculated weekly rates of stress-related and accidental injury emergency admission rates in England per 100 000 adolescents, stratified by gender and single year of age for each academic year, using published Office for National Statistics mid-year population estimates as the denominator.^25^ We used negative binomial regression to estimate incidence rate ratios (IRRs) for SRPs occurring in term versus holiday periods. IRRs were calculated separately for girls and boys of each single year of age, and adjusted for academic year (which was included in the model as a four-level factor variable). Negative binomial regression was used in preference to Poisson regression for all models because log-likelihood ratio testing indicated a superior fit (*P* < 0.001 in all instances).

We compared the characteristics of adolescents who were admitted with an SRP during term time or holidays in the most recent academic year (2017–2018), using chi-squared tests. For adolescents who had multiple SRPs within the academic year, one was randomly selected to be included in the analysis. We explored the following associations with ethnicity, area measures of deprivation and health-related factors: category of SRP, length of admission, previous unplanned hospital admissions, previous adversity-related admissions and chronic conditions.

We calculated the age-specific incidence of a first SRP in 2017–2018 by excluding those with a previous SRP admission since they were 11 years of age. Office for National Statistics mid-year population estimates were used as the denominator. To estimate the cumulative incidence of an SRP, we then added the age-specific incidence rates for 11- to 17-year-olds. All statistical analyses were carried out with StataMP for Windows version 16.

### Ethics statement

This study did not require ethical approval as it was an analysis of de-identified data. Routinely collected, de-identified patient data is made available for research purposes by the Health and Social Care Information Centre (NHS Digital) without individuals’ consent.^18^ This study used HES APC data, which was provided within the terms of a data-sharing agreement (agreement number DARS-NIC-393510-D6H1D-v4.14) to the researchers by NHS Digital.

## Results

The study included 571 388 school-aged adolescents who had 994 428 emergency admissions over the 4-year study period. Thirty-one per cent of all emergency admissions (305 491 of 994 428) were classified as an SRP, reflecting 39% (215 939 of 550 371) of emergency admissions in girls and 20% (89 552 of 444 057) in boys. In comparison, there were a total of 130 329 admissions relating to accidental injuries over the same period, reflecting 13% of all emergency admissions in adolescents: 8% in girls (42 153 of 550 371) and 20% in boys (88 176 of 444 057).

### Temporal and comparative trends in SRPs (2014–2015 to 2017–2018)

**Fig. 1:**
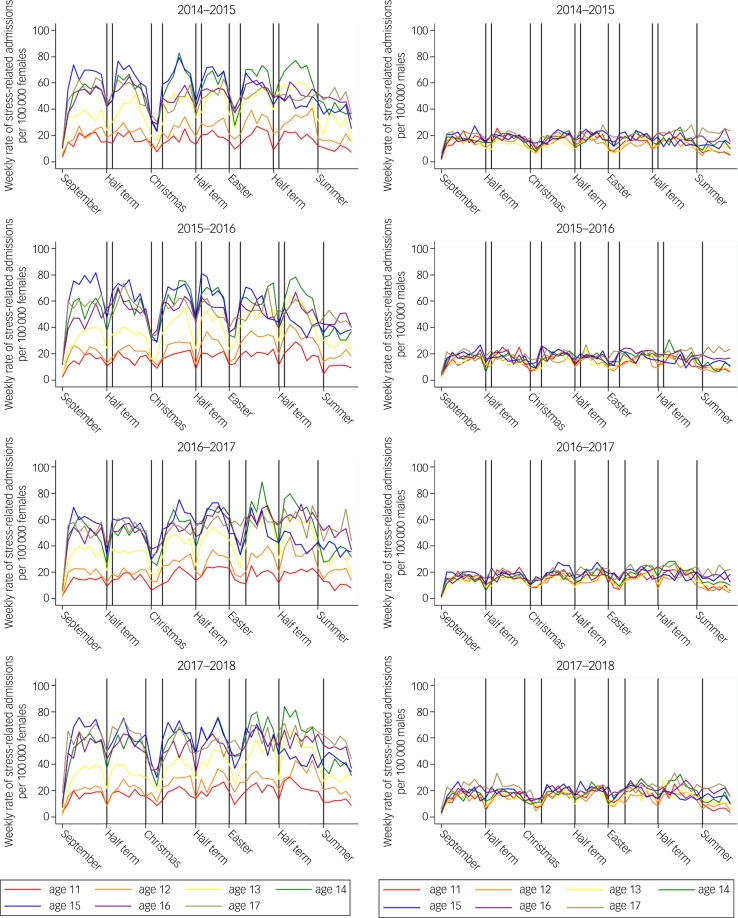
Weekly rates of stress-related presentations per 100 000 adolescent girls (left) and boys (right), by age and academic year (2014–2015 to 2017–2018).

**Fig. 2:**
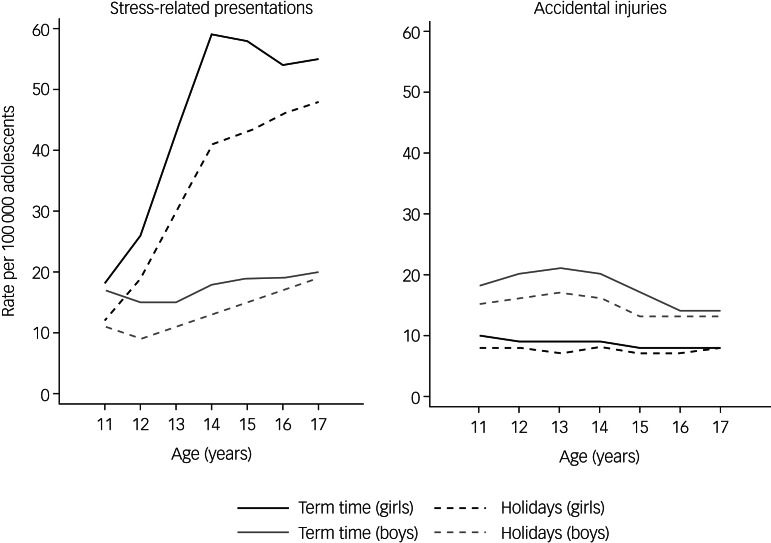
Overall mean weekly rate of stress-related presentations and accidental injury admissions per 100 000 adolescents, from 2014–2015 to 2017–2018**.**

Figure 1 shows the weekly, age-stratified rate of SRPs in adolescent girls and boys for each academic year. Mean term time and holiday rates of SRPs by academic year are given in Supplementary Table 6. Overall, term-time rates of SRPs were higher than holiday rates for girls and boys in all age groups, with the absolute rate difference being greatest for 14- and 15-year-old girls (Fig. 2). Relative differences were most marked in younger adolescent girls and boys, where term time was associated with a 50–60% increase in weekly rates of SRPs compared with holidays (Table 1). Overall rates of accidental injury admissions were also higher in term time than in holidays for adolescent girls and boys of all ages, except 17-year-old girls (Table 1). Mean term time and holiday rates of accidental injuries by academic year are given in Supplementary Table 7. Relative and absolute differences between rates of admission for accidental injuries in term compared with holidays were small for girls and boys (Fig. 2).

**Table 1:** Overall incidence rate ratio for stress-related presentations and accidental injury admissions per 100 000 adolescents in term time versus holidays, from 2014–2015 to 2017–2018

Gender	Age, years	Stress-related presentations			Accidental injury admissions		
		aIRR^a^	95% CI	aIRR^a^	95% CI
Girls	11	1.49	1.37	1.62	1.19	1.09	1.30
	12	1.41	1.29	1.54	1.13	1.05	1.23
	13	1.44	1.32	1.57	1.20	1.12	1.28
	14	1.45	1.35	1.56	1.20	1.12	1.29
	15	1.34	1.25	1.44	1.16	1.08	1.24
	16	1.18	1.12	1.25	1.09	1.01	1.17
	17	1.15	1.09	1.22	1.05	0.98	1.13
Boys	11	1.60	1.48	1.74	1.18	1.07	1.29
	12	1.55	1.43	1.68	1.24	1.13	1.35
	13	1.47	1.37	1.58	1.25	1.15	1.36
	14	1.41	1.31	1.53	1.31	1.21	1.42
	15	1.23	1.14	1.32	1.25	1.16	1.34
	16	1.12	1.05	1.19	1.11	1.04	1.19
	17	1.08	1.02	1.14	1.07	1.00	1.14

aIRR, adjusted incidence rate ratio.

a. Incidence rate ratios were adjusted for academic year (2014–2015, 2015–2016, 2016–2017, 2017–2018).

Over the 4-year study period, the burden of SRPs changed for some age groups. Compared with 2014–2015, in 2017–2018 there was an 18% increase in SRPs for 17-year-old girls (IRR of 1.18; 95% CI 1.10–1.27), a 12% increase for 13-year-old boys (IRR of 1.12; 95% CI 1.02–1.22) and a 10% increase for 16-year-old boys (IRR 1.10; 95% CI 1.01–1.19).

### Characteristics of adolescents with a SRP (2017–2018)

A total of 45 169 adolescents were admitted with an SRP in 2017–2018. The majority of SRPs were related to mental health problems or behavioural (drug/alcohol misuse, poisoning or self-harm) presentations (38%), or with abdominal pain (28%), headache or other pain (7%). There were small differences in the characteristics of adolescents with SRPs in term and holiday times (Table 2). Adolescents admitted in term time compared with holidays were on average younger, more frequently of White ethnicity, and girls (but not boys) were more frequently from households in less deprived areas. Presentation with pain-related stress symptoms did not differ between term time and holidays, but other types of somatic presentations and mental health problems/behavioural presentations did. Notably, self-harm and poisoning presentations were more commonly reported during term time than holidays for girls (33.3% of term-time SRPs versus 31.5% of holiday SRPs, *P* < 0.001). In contrast, self-harm and poisoning presentations were more commonly reported during holidays than term time for boys (15.7% of term-time SRPs versus 17.7% of holiday SRPs, *P* < 0.001). Drug and alcohol misuse presentations were less common during term time than during holidays for both boys and girls (Table 2). The majority of adolescents, particularly girls with term-time SRPs, did not have a history of being admitted with a previous SRP, adversity-related injury (relating to alcohol or drug use, or self-harm) or chronic conditions in the preceding 7-year period (Table 2). Among adolescents who had an SRP in 2017–2018, one in four (25.2%) had multiple SRPs in the year. Girls were more likely than boys to have multiple SRPs in a year (28.7% *v*. 18.2%, *P* < 0.001) (Supplementary Table 8).

**Table 2:** Characteristics of adolescents with stress-related presentations in holidays versus term time for the academic year 2017–2018, by gender

	Girls, *n* = 30 707			Boys, *n* = 14 462		
	Holidays	Term time		Holidays	Term time	
Characteristics	*n* (%)	*n* (%)	*P-*value	*n* (%)	*n* (%)	*P-*value
Age at start of academic year, years						
11	339 (6.1%)	1782 (7.1%)	<0.001	277 (10.5%)	1703 (14.4%)	<0.001
12	469 (8.4%)	2297 (9.1%)		288 (10.9%)	1476 (12.5%)	
13	691 (12.4%)	3473 (13.8%)		313 (11.9%)	1592 (13.5%)	
14	928 (16.6%)	4674 (18.6%)		378 (14.3%)	1816 (15.4%)	
15	949 (17.0%)	4451 (17.7%)		417 (15.8%)	1710 (14.5%)	
16	1051 (18.8%)	4107 (16.3%)		474 (18.0%)	1703 (14.4%)	
17	1151 (20.6%)	4345 (17.3%)		489 (18.6%)	1826 (15.4%)	
Ethnic group						
White	4261 (76.4%)	19 294 (76.8%)	0.05	1857 (70.5%)	8664 (73.3%)	0.03
Black/Black British	162 (2.9%)	668 (2.7%)		100 (3.8%)	393 (3.3%)	
Asian/Asian British	370 (6.6%)	1434 (5.7%)		240 (9.1%)	985 (8.3%)	
Other ethnic groups	275 (4.9%)	1291 (5.1%)		169 (6.4%)	636 (5.4%)	
Not known	510 (9.1%)	2442 (9.7%)		270 (10.2%)	1148 (9.7%)	
Quintile of deprivation, IMD						
1 (most deprived)	1554 (27.9%)	6668 (26.5%)	0.01	730 (27.7%)	3130 (26.5%)	0.20
2	1189 (21.3%)	5322 (21.2%)		554 (21.0%)	2460 (20.8%)	
3	1001 (18.0%)	4558 (18.1%)		444 (16.8%)	2166 (18.3%)	
4	973 (17.4%)	4244 (16.9%)		413 (15.7%)	1959 (16.6%)	
5 (least deprived)	861 (15.4%)	4337 (17.3%)		495 (18.8%)	2111 (17.9%)	
Length of stress-related hospital stay, nights						
0	2725 (48.9%)	11 489 (45.7%)	<0.001	1416 (53.7%)	6416 (54.2%)	0.60
1	1720 (30.8%)	8426 (33.5%)		776 (29.4%)	3543 (30.0%)	
2	439 (7.9%)	2278 (9.1%)		196 (7.4%)	812 (6.9%)	
≥3	694 (12.4%)	2936 (11.7%)		248 (9.4%)	1055 (8.9%)	
Category of stress-related presentation						
Mental health and behavioural	2393 (42.9%)	10 671 (42.5%)	<0.001	867 (32.9%)	3144 (26.6%)	<0.001
Mental health (including sleep disorders)	422 (7.6%)	1718 (6.8%)		178 (6.8%)	718 (6.1%)	
Drug/alcohol misuse	218 (3.9%)	584 (2.3%)		223 (8.5%)	566 (4.8%)	
Self-harm/poisoning/other	1753 (31.4%)	8369 (33.3%)		466 (17.7%)	1860 (15.7%)	
Pain-related	1903 (34.1%)	8554 (34.0%)	0.65	866 (32.9%)	4550 (38.5%)	0.83
Abdominal/pelvic pain	1555 (27.9%)	6953 (27.7%)		631 (23.9%)	3395 (28.7%)	
Headache	233 (4.2%)	1111 (4.4%)		190 (7.2%)	904 (7.6%)	
Other pain	115 (2.1%)	490 (1.9%)		45 (1.7%)	251 (2.1%)	
Other somatic presentations	1282 (23.0%)	5904 (23.5%)	<0.001	903 (34.3%)	4132 (34.9%)	<0.001
Circulatory/respiratory signs	365 (6.5%)	1500 (6.0%)		305 (11.6%)	1247 (10.5%)	
Digestive symptoms	147 (2.6%)	470 (1.9%)		92 (3.5%)	374 (3.2%)	
Skin symptoms	70 (1.3%)	319 (1.3%)		66 (2.5%)	272 (2.3%)	
Nervous/musculoskeletal symptoms	42 (0.8%)	206 (0.8%)		28 (1.1%)	130 (1.1%)	
Cognitive symptoms	55 (1.0%)	264 (1.1%)		46 (1.7%)	191 (1.6%)	
Malaise/fatigue/syncope	240 (4.3%)	984 (3.9%)		173 (6.6%)	690 (5.8%)	
Other/general symptoms	363 (6.5%)	2 161 (8.6%)		193 (7.3%)	1228 (10.4%)	
Healthcare history in the preceding 7 years						
Unplanned hospital admissions						
0	3368 (60.4%)	15 663 (62.3%)	0.004	1841 (69.8%)	8253 (69.8%)	0.88
1–2	951 (17.1%)	4342 (17.3%)		417 (15.8%)	1915 (16.2%)	
3–4	885 (15.9%)	3601 (14.3%)		267 (10.1%)	1195 (10.1%)	
≥5	374 (6.7%)	1523 (6.1%)		111 (4.2%)	463 (3.9%)	
Any chronic condition recorded	1610 (28.9%)	6569 (26.1%)	<0.001	477 (18.1%)	2121 (17.9%)	0.85
Injury indicating adversity						
Violence	58 (1.0%)	186 (0.7%)	0.02	26 (1.0%)	93 (0.8%)	0.30
Drug/alcohol use	761 (13.6%)	3 045 (12.1%)	0.002	118 (4.5%)	451 (3.8%)	0.11
Self-harm	883 (15.8%)	3 665 (14.6%)	0.02	125 (4.7%)	526 (4.5%)	0.51

*P*-values relate to a chi-squared test comparing the proportion of presentations in term time versus holidays within the presented subgroups. IMD, Index of Multiple Deprivation.

### Estimated cumulative incidence of SRPs (2017–2018)

In 2017–2018, 30 707 girls and 14 462 boys were admitted with an SRP, equating to 1.5% of adolescent girls and 0.65% of adolescent boys (see Supplementary Table 9). The majority of adolescents (*n* = 36 254, 80%) had no record of a previous SRP during adolescence (i.e. from the age of 11 years). By adding the age-specific rates of a first SRP, we estimated the cumulative incidence between 11 and 17 years to be approximately 7.9% of girls and 4.1% of boys (see Supplementary Table 9). These figures do not account for temporal trends in stress-related conditions, as reported above.

## Discussion

### Main findings

We found that 7.9% of girls (1 in 13) and 4.1% of boys (1 in 25) were admitted to hospital with an SRP between the ages of 11 and 17 years. Rates of SRPs were significantly higher during term time than holidays for adolescents of all ages, with rates being highest in girls (particularly 14- and 15-year-olds), and with less variation by age in boys. The frequency and healthcare burden of SRPs was substantial, particularly for girls. SRPs accounted for 39% of all emergency admissions in girls during the study period and 20% in boys. Rates of admissions for SRPs increased for some groups over the study period (i.e. girls aged 17 years and boys aged 13 and 16 years).

### Strengths and limitations

The key strength of our study is that it used a longitudinal hospital admissions data-set that includes the date of admission. This allowed us to evaluate the timing of SRPs in relation to term time and holidays across academic years, and to estimate the cumulative incidence of an SRP among adolescents of secondary school age (i.e. 11–17 years). As the hospital administrative data includes all admissions to NHS hospitals in England, our results are broadly representative of adolescents in English schools. A limitation of administrative hospital admissions data is that it can only capture a subset of SRPs, as many children will seek care from other health settings (such as general practice). It is also likely that many children will not have contact with any health services at all; for example, a 2014 cross-sectional survey of adults found that the majority (75.6%) had no contact with medical services following non-suicidal self-harm.^26^ Contacts with health services also vary by demographic factors; for example, women are more likely to have contact with health services following non-suicidal self-harm,^26^ and ethnic minorities with common mental health disorders are less likely to see their general practitioner than their White peers.^27^ Therefore, it is possible that the higher rates of SRPs among girls, particularly those who were White and less deprived, may be partially attributable to differences in health-seeking behaviour and equity of access to hospital healthcare.

A further strength of our study is the development of a code list for SRPs that builds on definitions applied by existing literature; however, data permissions did not allow validation of codes through case note review. This means that some admissions may have been misclassified as stress-related because of the absence of a recorded surgical or medical cause, which may have led to an overestimation of SRPs. Our chosen comparison group (accidental injuries) could also be misclassified, as some injuries may reflect risk-taking or self-harming behaviour not recorded by clinicians and therefore not recorded in coded administrative hospital records.^23^ Nevertheless, the differences between term time and holiday admissions for SRPs were greater than for accidental injury admissions, particularly for girls.

### Context of our findings in relation to what is known

Our results, based on analysis of whole-population administrative data sources, confirm patterns reported in surveys of school-aged adolescents, using self-report questionnaires^7^ and psychometric measures.^6^ The high proportion of presentations related to pain, mental health problems and self-harm are consistent with previous studies.^6,7,11^ The preponderance of girls and higher rates in mid-to-late adolescence are consistent with previous studies.^6^^,^^9^ We found no previous studies that directly compared rates of SRPs in school term with holiday time. Previous research has found higher rates of suicide during term time compared with holidays among secondary school students.^28^^,^^29^

### Implications for policy, practice and research

SRPs to hospitals might be an early indicator of vulnerability and represent an opportunity to tackle the early signs of mental health issues^15^ through effective and appropriate mental health interventions, which could ultimately reduce the likelihood of clinical conditions developing in later life. The substantial healthcare burden of SRPs among adolescents admitted to hospital suggests that there is a need for mental health training for all paediatric staff in hospitals and for better access to specialist mental healthcare for children being treated in hospitals, such as mental health liaison services.

SRPs to hospital are the tip of the iceberg of a very common and complex problem among adolescents, particularly girls.^7,14^ Our study was restricted to hospital admissions, whereas most emergency healthcare presentations by adolescents are to the emergency department or primary care. Administrative data from emergency departments have not been adequately coded in England to investigate SRPs by using the approach in our current study. Development and validation of a coding list for SRPs in children and young people in primary care data has not been done, to our knowledge, but would be valuable. Given the high burden of self-reported stress-related symptoms in adolescence, primary care responses are an important point of intervention.

Higher rates of SRPs in term time than holidays, particularly for girls, suggest that the school environment could be a contributing factor. School-based interventions to prevent and reduce mental health problems are widespread, although evidence of effectiveness is limited, partly because of the need to account for the emergence of underlying mental health problems in adolescence.^16,30^ Our findings make clear the need to consider short- and long-term effects on healthcare costs when evaluating interventions in schools. The differences in associations between SRPs during term time and holidays that we observed by age and gender also suggest that schools may need to take targeted approaches that account for different stages of neurobiological development during adolescence, to mediate the role of stress on risk and resilience and prevent and reduce mental health problems among students.^30,^^31^ Our findings only measure the immediate impact of adolescent SRPs on healthcare, and do not examine the long-term health consequences. Policy decisions need to consider healthcare consequences and costs in decisions about returns on investments to promote a healthy school environment.

Among adolescents who had an SRP in 2017–2018, a quarter had multiple SRPs in the year. Repeated SRPs may be an indicator of unmet need that requires targeted intervention. Future research could explore whether there are distinct subgroups of children who have multiple SRPs, using methods such as latent class or trajectory analysis. Our dichotomous categorisation of term and holiday time did not allow the role of seasonal changes on SRPs to be examined. Future work could also examine differences between term and holiday time, accounting for season.

Our ecological analysis assumes that all children aged 11–17 years were enrolled in school. Children who were not enrolled in school or who were home schooled could not be identified in administrative hospital data because this information is not recorded. It was also not possible to explore whether SRPs were related to taking public examinations because, during the study period (2014–2015 to 2017–2018), there was considerable overhaul of the examination system in England, with a graduated move from modular to linear assessments for public examinations at ages 15 and 17 years (Key Stages 4 and 5). Individual-level information about school enrolment, year group and examination entries would be required to explore this question robustly. Future research in this area could benefit from improved data infrastructure being established for England through the Education and Child Health Insights from Linked Data (ECHILD) Database.^32^ The ECHILD Database links hospital and education administrative for all children in England, and will be accessible to researchers in 2022. The ECHILD Database will enable future exploration of how SRPs vary over time (including during examination years or following disruptions to educational careers, such as school transfers or exclusions) and according to school characteristics, taking into account adolescents’ past health and education histories. Variation between schools could prompt qualitative evaluation of school environments to understand differences in practice and support randomised intervention studies. Linked administrative data presents a unique opportunity to measure and monitor SRPs among adolescents,^32^ and future enhancements of the ECHILD Database should consider adding primary care and mental health service records, which would provide more granular information about how and where adolescents present with stress-related symptoms.

## Data Availability

This study uses NHS Hospital Episode Statistics data and was provided within the terms of a data-sharing agreement (number DARS-NIC-393510-D6H1D-v4.14) to the researchers by the Health and Social Care Information Centre (NHS Digital). The data do not belong to the authors and may not be shared by the authors, except in aggregate form for publication. Data can be obtained by submitting a data request through the NHS Digital Data Access Request Service.

## Supplementary material

For supplementary material accompanying this paper visit http://dx.doi.org/10.1192/bjo.2021.1058. click here to view supplementary material
