# Supplementary material for: Hospital admissions for stress-related presentations among school-aged adolescents during term time versus holidays in England: weekly time series and retrospective cross-sectional analysis
Source: BJPsych Open. 2021 Nov 19;7(6):e215. doi: 10.1192/bjo.2021.1058 (PMC8612011; doi:10.1192/bjo.2021.1058)
Supplement: Supplementary file 1 [file S2056472421010589sup001.docx]

**Supplementary Material 1**

Hospital Episode Statistics Admitted Patient Care study data extract

|  | **Adolescents** | **Admissions** |  |  |  |
| --- | --- | --- | --- | --- | --- |
| Eligible population: Adolescents resident in England aged 11 to 17 years with 1+ emergency admission between 1^st^ September 2014 and 31^st^ August 2018 | 591 576 | 1 313 642 |  |  |  |
| Exclude: |  |  |  | **Adolescents** | **Admissions** |
| (a) Individuals of unknown sex |  |  | → | 141 |  |
| (b) Maternity admissions |  |  | → | 7 725 | 33 502 |
| (c) Adolescents who died in hospital |  |  | → | 430 |  |
| (d) Admissions for abdominal pain with a likely cause |  |  | → | 4 959 | 15 544 |
| (e) Individuals who no longer meet the eligibility criteria of 1+ emergency admission following the application of exclusion criteria (a) to (d) |  |  | → | 6 933 |  |
|  |  |  |  |  |  |
| Study sample | 571 388 | 994 428 |  |  |  |
| Stress-related presentations | 171 013 | 305 491 |  |  |  |
| Accidental injuries comparison group | 106 646 | 130 329 |  |  |  |

As one of the study objectives was to explore sex differences in rates of SRPs, we also excluded adolescents with unrecorded sex and any pregnancy-related admissions. We excluded adolescents who died in hospital because retrospective clinical coding at the time of hospital discharge may systematically differ for patients who died compared to those who are discharged alive.

**Supplementary Material 2**

School term and holiday periods in England, by academic year (2013/14 to 2017/18)

| **Academic year** | **2013/14** | **2014/15** | **2015/16** | **2016/17** | **2017/18** |
| --- | --- | --- | --- | --- | --- |
| Autumn Term | 03/09/13 to 20/12/13 | 02/09/14 to 19/12/14 | 02/09/15 to 18/12/15 | 05/09/16 to 21/12/16 | 04/09/17 to 15/12/17 |
| Half term - Autumn | 28/10/13 to 01/11/13 | 23/10/14 to 31/10/14 | 26/10/15 to 30/10/15 | 24/10/16 to 28/10/16 | 23/10/17 to 27/10/17 |
| Christmas holiday | 21/12/13 to 05/01/14 | 20/12/14 to 04/01/15 | 19/12/15 to 04/01/16 | 22/12/16 to 03/01/17 | 16/12/17 to 01/01/18 |
| Spring Term | 06/01/14 to 04/04/14 | 05/01/15 to 27/03/15 | 05/01/16 to 24/03/16 | 04/01/17 to 31/03/17 | 02/01/18 to 29/03/18 |
| Half term - Spring | 17/02/14 to 21/02/14 | 16/02/15 to 20/02/15 | 15/02/16 to 19/02/16 | 13/02/17 to 17/02/17 | 12/02/18 to 16/02/18 |
| Easter holiday | 05/04/14 to 21/04/14 | 28/03/15 to 12/04/15 | 25/03/16 to 10/04/16 | 01/04/17 to 17/04/17 | 30/03/18 to 15/04/18 |
| Summer Term | 22/04/14 to 23/07/14 | 13/04/15 to 21/07/15 | 11/04/16 to 22/07/16 | 18/04/17 to 21/07/17 | 16/04/18 to 27/07/18 |
| Half term -Summer | 26/05/14 to 30/05/14 | 25/05/15 to 29/05/15 | 30/05/16 to 03/06/16 | 29/05/17 to 02/06/17 | 28/05/18 to 01/06/18 |
| Summer holiday | 24/07/14 to 01/09/14 | 22/07/15 to 02/09/15 | 23/07/16 to 04/09/16 | 22/07/17 to 03/09/17 | 28/07/18 to 02/09/18 |

Supplementary Material 2 specifies the time periods categorised as term-time (grey shading) versus holidays in this study based on a review of school timetables published online by local authorities.

**Supplementary Material 3**

International Classification of Diseases and Related Health Problems version 10 (ICD-10) code list for stress-related presentations

| **Category** | **Group** | **ICD-code** | **ICD-10 Description** |
| --- | --- | --- | --- |
| Pain-related presentations | Abdominal/pelvic pain | R10 ^1^ | Abdominal and pelvic pain |
|  | Headache | R51 | Headache |
|  |  | G442 | Tension-type headache |
|  | Other pain | M54 | Panniculitis affecting regions of neck and back |
|  |  | M626 | Muscle strain |
|  |  | M796 | Pain in limb |
|  |  | R52 | Acute pain |
| Other somatic presentations | Circulatory/respiratory signs | R00 | Abnormalities of heart beat |
|  |  | R03 | Abnormal blood pressure reading, without diagnosis |
|  |  | R05 | Cough |
|  |  | R06 | Abnormalities of breathing |
|  |  | R07 | Pain in throat and chest |
|  | Digestive symptoms | R11-14 | Nausea and vomiting, Heartburn, Dysphagia, Flatulence and related conditions |
|  |  | R194 | Change in bowel habit |
|  | Skin symptoms | R20-21 | Disturbances of skin sensation, rash and other nonspecific skin eruption |
|  |  | R231 | Pallor |
|  |  | R234 | Changes in skin texture |
|  |  | R238 | Other and unspecified skin changes |
|  | Nervous/musculoskeletal symptoms | R25 | Abnormal involuntary movements |
|  |  | R26 | Abnormalities of gait and mobility |
|  |  | R27 | Other lack of co-ordination |
|  |  | R292-294 | Abnormal reflex, Abnormal posture, Clicking hip |
|  |  | R298 | Other and unspecified signs and symptoms involving nervous and musculoskeletal systems |
|  | Cognitive symptoms | R400-401 | Somnolence, Stupor |
|  |  | R41-42 | Other symptoms and signs involving cognitive functions and awareness, Dizziness and giddiness |
| Other somatic presentations (continued) | Malaise/Fatigue/Syncope | R53 | Malaise and fatigue |
|  |  | R55 | Syncope and collapse |
|  | Other/general symptoms | R44 | Other symptoms and signs involving general sensations and perceptions |
|  |  | R45 | Symptoms and signs involving emotional state |
|  |  | R46 | Symptoms and signs involving appearance and behaviour |
|  |  | R47 | Dysphasia and aphasia |
|  |  | R49 | Voice disturbances |
|  |  | Z563-564 | Stressful work schedule, Discord with boss and workmates |
|  |  | Z711 | Person with feared complaint in whom no diagnosis is made |
|  |  | Z733 | Stress, not elsewhere classified |
| Mental health & behavioural presentations | Anxiety/Depression | F31 | Bipolar affective disorder |
|  |  | F320 | Mild depressive episode |
|  |  | F321 | Moderate depressive episode |
|  |  | F322 | Severe depression without psychotic symptoms |
|  |  | F323 | Severe depression with psychotic symptoms |
|  |  | F328 | Other depressive episodes |
|  |  | F329 | Depressive episode, unspecified |
|  |  | F330 | Recurrent depressive disorder, current episode mild |
|  |  | F331 | Recurrent depressive disorder, current episode moderate |
|  |  | F332 | Recurrent depressive disorder, current episode severe without psychotic symptoms |
|  |  | F333 | Recurrent depressive disorder, current episode severe with psychotic symptoms |
|  |  | F338 | Other recurrent depressive disorders |
|  |  | F339 | Recurrent depressive disorder, unspecified |
|  |  | F341 | Dysthymia |
|  |  | F40 | Agoraphobia |
|  |  | F410 | Social phobias |
|  |  | F411 | Generalized anxiety disorder |
|  |  | F412 | Mixed anxiety and depressive disorder |
| Mental health & behavioural presentations (cont.) | Anxiety/Depression (cont.) | F43 | Other mixed anxiety disorder |
|  | Mental health | F20-F29 | Schizophrenia, schizotypal and delusional disorders |
|  |  | F30-F39 | Mood disorders |
|  |  | F40-F49 | Neurotic, stress-related and somatoform disorders |
|  |  | F50-F59 | Behavioural syndromes associated with physiological disturbances and physical factors |
|  |  | F60-F69 | Disorders of adult personality and behaviour |
|  |  | F90-F98 | Behavioural and emotional disorders with onset usually occurring in childhood and adolescence |
|  |  | F99 | Unspecified mental disorder |
|  | Obsessive-compulsive / Dissociative / Eating disorders | F42 | Obsessive-compulsive disorder |
|  |  | F44 | Dissociative [conversation] disorders |
|  |  | F45 | Somatoform disorders |
|  |  | F50 | Eating disorders |
|  | Sleep disorders | F51 | Nonorganic sleep disorders |
|  |  | G47 | Disorders of initiating and maintaining sleep [insomnias] |
|  | Drug / Alcohol abuse | F10-F19 | Mental and behavioural disorders due to psychoactive substance use |
|  |  | F55 | Abuse of non-dependence-producing substances |
|  |  | R780 | Finding of alcohol in blood |
|  |  | R781-R785 | Findings of drugs and other substances, not normally found in blood |
|  |  | T51 | Toxic effect of alcohol |
|  |  | Y15 ^2^ | Poisoning by drugs, medicaments and biological substances |
|  |  | Y90 ^2, 3^ | Evidence of alcohol involvement determined by blood alcohol level |
|  |  | Y91 ^2, 3^ | Evidence of alcohol involvement determined by level of intoxication |
|  |  | Z040 ^2, 3^ | Blood-alcohol and blood-drug test |
|  |  | Z502 ^2, 3^ | Alcohol rehabilitation |
|  |  | Z503 ^2, 3^ | Drug rehabilitation |
|  |  | Z714 ^2, 3^ | Alcohol abuse counselling and surveillance |
|  |  | Z715 ^2, 3^ | Drug abuse counselling and surveillance |
|  |  | Z721 ^2, 3^ | Alcohol use |
| Mental health & behavioural presentations (cont.) | Drug / Alcohol abuse (cont.) | Z722 ^2, 3^ | Drug use |
|  |  | Z915 ^2, 4^ | Personal history of self-harm |
|  | Self-harm / Poisoning / Cutting | T36-50 ^3^ | Poisoning by drugs, medicaments and biological substances |
|  |  | Y10-14 ^2^ | Poisoning [drugs], undetermined intent |
|  |  | Y16-19 ^2^ | Poisoning [chemicals], undetermined intent |
|  |  | S00 ^3^ | Superficial injury of scalp |
|  |  | S10 ^3^ | Superficial injury of neck |
|  |  | S20 ^3^ | Superficial injury of thorax |
|  |  | S30 ^3^ | Superficial injury of lower back and pelvis |
|  |  | S40 ^3^ | Superficial injury of shoulder and upper arm |
|  |  | S50 ^3^ | Superficial injury of forearm |
|  |  | S60 ^3^ | Superficial injury of wrist and hand |
|  |  | S70 ^3^ | Superficial injury of hip and thigh |
|  |  | S80 ^3^ | Superficial injury of lower leg |
|  |  | S90 ^3^ | Superficial injury of ankle and foot |
|  |  | X60-63 ^2^ | Intentional self-poisoning (drugs) |
|  |  | X64-X69 ^2^ | Intentional self-harm (self-poisoning) |
|  |  | X70-X84 2 | Intentional self-harm (hanging, drowning, firearm, explosive material, fire, steam, sharp/blunt object, jumping, crashing motor vehicle, other) |
|  |  | Z642 ^2^ | Intentional self-poisoning by and exposure to other and unspecified drugs, medicaments and biological substances |
|  |  | Z915 ^2, 4^ | Personal history of self-harm |

ICD-10 = International Classification of Diseases and Related Health Problems version 10. Supplementary Material 3 outlines the ICD-10 code list that was developed as part of this study to identify a stress-related presentation in Hospital Episode Statistics Admitted Patient Care (HES APC) data based on information recorded in the diagnosis fields. In HES APC up to 20 diagnosis fields can be recorded per admission using ICD-10 codes. An admission was categorised as a stress-related presentation if an ICD-10 code listed in Supplementary Material 3 was recorded in the primary diagnostic position, or in any diagnostic position for some mental health and behavioural presentations, as indicated. ^1^ Admissions with a primary diagnostic code of R10 (Abdominal and pelvic pain) were not categorised as a stress-related presentation if a medical or surgical cause was indicated by an operation or subsidiary diagnostic code for the same admission (as detailed in Supplementary Material 4). ^2^ An admission was categorised as a stress-related presentation if this ICD-10 code was recorded in any diagnostic coding position. ^3^ An admission was categorised as a stress-related presentation if this ICD-10 code was recorded in the primary diagnostic coding position and one of the following self-harm codes were also recorded in another diagnostic position: X60-63 (Intentional self-poisoning (drugs)), X64-X69 (Intentional self-harm (self-poisoning)), X70-X84 (Intentional self-harm (hanging, drowning, firearm, explosive material, fire, steam, sharp/blunt object, jumping, crashing motor vehicle, other)), Z642 (Intentional self-poisoning by and exposure to other and unspecified drugs, medicaments and biological substances) or Z915 (personal history of self-harm). ^4^ For admissions that included Z915 (personal history of self-harm), where the diagnostic codes reflected more than one category (e.g., drug/alcohol abuse and self-harm presentations), the presentation was classified as Self-harm/ Poisoning / Cutting only, such that the groups were mutually exclusive.

**Supplementary Material 4**

Diagnostic and operation codes indicating a medical or surgical cause for presentations of abdominal pain

|  | **Code** | **Description** |
| --- | --- | --- |
| Diagnosis | N832 | Other and unspecified ovarian cysts |
|  | A099 | Gastroenteritis and colitis of unspecified origin |
|  | K529 | Noninfective gastroenteritis and colitis, unspecified |
|  | E282 | Polycystic ovarian syndrome |
|  | N390 | Urinary tract infection, site not specified |
|  | K589 | Irritable bowel syndrome without diarrhoea |
| Operation | Y752 | Laparoscopic approach to the abdominal cavity not elsewhere classified |
|  | H013 | Emergency excision of normal appendix |
|  | H029 | Unspecified excision of appendix |
|  | H012 | Emergency excision of normal appendix not elsewhere classified |
|  | H019 | Unspecified emergency excision of appendix |
|  | H021 | Interval appendectomy |
|  | H023 | Prophylactic appendectomy NEC |
|  | H024 | Incidental appendectomy |
|  | H028 | Other specified excision of appendix |
|  | H011 | Emergency excision of abnormal appendix and drainage HFQ |

In Hospital Episode Statistics Admitted Patient Care data, up to 20 diagnosis fields and 24 operation fields can be recorded per admission using standardised coding systems (International Classification of Diseases and Related Health Problems version 10 (ICD-10) codes for diagnoses and Office of Population Censuses and Surveys version 4 (OPCS-4) for operations). Admissions with a primary diagnostic code of R10 (Abdominal and pelvic pain) were not categorised as a stress-related presentation if a medical or surgical cause was indicated by a subsidiary diagnostic code or any operation code listed in Supplementary Material 4 recorded for the same admission.

**Supplementary Material 5**

International Classification of Diseases and Related Health Problems version 10 (ICD-10) code list for accidental injuries

| **ICD-10 code** | **ICD-10 Description** |
| --- | --- |
| V | Accidents |
| W0-W9 | Falls, exposure to mechanical forces, accidental drowning, accidental threats to breathing, exposure to electric current, extreme temperature or pressure |
| X0-X5 | Exposure to smoke, fire and flames, contact with heat, venomous animals and plants, forces of nature, accidental poisoning, overexertion, other or unspecified accidental exposure |

Supplementary Material 5 outlines the ICD-10 code list that was used to identify accidental injuries admissions in Hospital Episode Statistics Admitted Patient Care (HES APC) data based on information recorded in the diagnosis fields. In HES APC up to 20 diagnosis fields can be recorded per admission using ICD-10 codes. An admission was categorised as an accidental injury if an ICD-10 code listed in Supplementary Material 5 was recorded in any diagnostic position. Admissions where these accidental injury codes were identified were not categorised as stress-related presentations such that the two groups were mutually exclusive.

**Supplementary Material 6**

Mean weekly rates (per 100,000 adolescents) of stress-related presentations occurring in term or holiday time by academic year, age and sex

| **Sex** | **Age (years)** | **Mean weekly rates of stress-related presentations per 100,000 adolescents** | | | | | | | |
| --- | --- | --- | --- | --- | --- | --- | --- | --- | --- |
|  |  | **2014/15** | | **2015/16** | | **2016/17** | | **2017/18** | |
|  |  | **Term** | **Holiday** | **Term** | **Holiday** | **Term** | **Holiday** | **Term** | **Holiday** |
| Girls | 11 | 18 | 13 | 18 | 12 | 18 | 12 | 19 | 12 |
|  | 12 | 26 | 18 | 26 | 19 | 27 | 19 | 26 | 18 |
|  | 13 | 43 | 30 | 42 | 29 | 44 | 30 | 42 | 28 |
|  | 14 | 58 | 42 | 59 | 41 | 57 | 39 | 60 | 41 |
|  | 15 | 58 | 42 | 60 | 45 | 55 | 41 | 59 | 45 |
|  | 16 | 51 | 44 | 53 | 46 | 56 | 47 | 56 | 45 |
|  | 17 | 50 | 45 | 55 | 46 | 55 | 49 | 60 | 51 |
| Boys | 11 | 17 | 10 | 16 | 11 | 17 | 10 | 17 | 10 |
|  | 12 | 14 | 9 | 14 | 10 | 15 | 9 | 15 | 9 |
|  | 13 | 14 | 10 | 15 | 11 | 15 | 10 | 16 | 11 |
|  | 14 | 17 | 13 | 18 | 13 | 18 | 13 | 19 | 13 |
|  | 15 | 19 | 15 | 18 | 14 | 19 | 16 | 19 | 15 |
|  | 16 | 18 | 17 | 19 | 16 | 18 | 16 | 20 | 17 |
|  | 17 | 20 | 20 | 19 | 19 | 20 | 18 | 22 | 19 |

Age was defined as age at the start of the academic year. Office for National Statistics mid-year population estimates were used as denominator data.

**Supplementary Material 7**

Mean weekly rates (per 100,000 adolescents) of accidental injury admissions occurring in term or holiday time by academic year, age and sex

| **Sex** | **Age (years)** | **Mean weekly rates of accidental injury admissions per 100,000 adolescents** | | | | | | | |
| --- | --- | --- | --- | --- | --- | --- | --- | --- | --- |
|  |  | **2014/15** | | **2015/16** | | **2016/17** | | **2017/18** | |
|  |  | **Term** | **Holiday** | **Term** | **Holiday** | **Term** | **Holiday** | **Term** | **Holiday** |
| Girls | 11 | 10 | 8 | 10 | 9 | 9 | 8 | 9 | 7 |
|  | 12 | 9 | 8 | 9 | 7 | 9 | 8 | 9 | 8 |
|  | 13 | 9 | 7 | 8 | 7 | 9 | 8 | 9 | 6 |
|  | 14 | 9 | 8 | 9 | 8 | 10 | 8 | 9 | 7 |
|  | 15 | 8 | 7 | 8 | 7 | 8 | 7 | 9 | 7 |
|  | 16 | 7 | 6 | 8 | 7 | 8 | 8 | 8 | 7 |
|  | 17 | 8 | 8 | 8 | 8 | 8 | 8 | 9 | 8 |
| Boys | 11 | 18 | 16 | 17 | 16 | 18 | 16 | 17 | 12 |
|  | 12 | 21 | 17 | 19 | 16 | 21 | 17 | 18 | 13 |
|  | 13 | 21 | 16 | 21 | 17 | 22 | 19 | 20 | 15 |
|  | 14 | 20 | 15 | 20 | 16 | 21 | 17 | 19 | 14 |
|  | 15 | 17 | 13 | 16 | 12 | 17 | 15 | 16 | 12 |
|  | 16 | 14 | 13 | 13 | 12 | 13 | 13 | 14 | 12 |
|  | 17 | 14 | 13 | 14 | 14 | 14 | 13 | 14 | 13 |

Age was defined as age at the start of the academic year. Office for National Statistics mid-year population estimates were used as denominator data.

**Supplementary Material 8**

Summary of stress-related presentations (SRPs) among adolescents aged 11-17 years in England in 2017/18, by sex

| \|  \| **Overall**  **N = 45 169** \| **Girls**  **N = 30 707** \| **Boys**  **N = 14 462** \| \| --- \| --- \| --- \| --- \| \| Range \| 1 to 68 \| 1 to 68 \| 1 to 31 \| \| Mean \| 1.57 \| 1.68 \| 1.36 \| \| Median \| 1 \| 1 \| 1 \| \| % with 1+ SRP \| 25.2% \| **28.7%** \| **18.2%** \| |
| --- | --- | --- | --- | --- | --- | --- | --- | --- | --- | --- | --- | --- | --- | --- | --- | --- | --- | --- | --- | --- |
|  |
| SRP = Stress-related presentation. Bold indicates *P*<0.001. |

**Supplementary Material 9**

Age-specific and estimated cumulative incidence of a stress-related presentation (SRP) among adolescents aged 11-17 years in England in 2017/18, by sex

| \| **Sex** \| **Age (years)** \| **Population denominator** \| **Total with a first SRP** \| **Age-specific incidence** \| **Estimated cumulative incidence *** \| \| --- \| --- \| --- \| --- \| --- \| --- \| \| Girls \| 11 \| 319 229 \| 2 049 \| 0.64 \| 0.64 \| \| 12 \| 306 248 \| 2 475 \| 0.81 \| 1.46 \| \| 13 \| 301 164 \| 3 430 \| 1.14 \| 2.60 \| \| 14 \| 292 004 \| 4 337 \| 1.49 \| 4.11 \| \| 15 \| 287 102 \| 3 942 \| 1.37 \| 5.50 \| \| 16 \| 295 689 \| 3 495 \| 1.18 \| 6.70 \| \| 17 \| 302 735 \| 3 713 \| 1.23 \| 7.94 \| \| Boys \| 11 \| 335 137 \| 1 927 \| 0.57 \| 0.57 \| \| 12 \| 321 159 \| 1 659 \| 0.52 \| 1.09 \| \| 13 \| 315 811 \| 1 734 \| 0.55 \| 1.65 \| \| 14 \| 307 468 \| 1 930 \| 0.63 \| 2.28 \| \| 15 \| 302 504 \| 1 820 \| 0.60 \| 2.88 \| \| 16 \| 310 035 \| 1 810 \| 0.58 \| 3.47 \| \| 17 \| 320 527 \| 1 933 \| 0.60 \| 4.08 \| |
| --- | --- | --- | --- | --- | --- | --- | --- | --- | --- | --- | --- | --- | --- | --- | --- | --- | --- | --- | --- | --- | --- | --- | --- | --- | --- | --- | --- | --- | --- | --- | --- | --- | --- | --- | --- | --- | --- | --- | --- | --- | --- | --- | --- | --- | --- | --- | --- | --- | --- | --- | --- | --- | --- | --- | --- | --- | --- | --- | --- | --- | --- | --- | --- | --- | --- | --- | --- | --- | --- | --- | --- | --- | --- | --- | --- | --- | --- | --- |
| Age was defined as age at the start of the academic year. Office for National Statistics mid-year population estimates were used as denominator data. *The estimated cumulative incidence was calculated by summing the age-specific incidences. The figures presented in Supplementary Material 9 are rounded to 2 decimal places and so the cumulative incidence may not exactly equal the sum of the preceding age-specific incidences. |
